# Dose-response Relationship of Serum Uric Acid with Metabolic Syndrome and Non-alcoholic Fatty Liver Disease Incidence: A Meta-analysis of Prospective Studies

**DOI:** 10.1038/srep14325

**Published:** 2015-09-23

**Authors:** Zhengtao Liu, Shuping Que, Lin Zhou, Shusen Zheng

**Affiliations:** 1 Key Laboratory of Combined Multi-Organ Transplantation, Ministry of Public Health and Key Laboratory of Organ Transplantation of Zhejiang Province, Hangzhou, 310003, Zhejiang province, China; 2 Collaborative Innovation Center for Diagnosis and Treatment of Infectious Diseases, Hangzhou, 310003, China; 3 Division of Hepatobiliary and Pancreatic Surgery, Department of Surgery, First Affiliated Hospital, School of Medicine, Zhejiang University, Hangzhou, 310003, China; 4 Department of Pediatrics, Women and children's hospital of Guangxi, Nanning, 530005, Guangxi province, China

## Abstract

Emerging evidence has shown that serum uric acid (SUA) elevation might cause metabolic derangements, including metabolic syndrome (MetS) and non-alcoholic fatty liver disease (NAFLD); however, magnitude of the risk has not been quantified. We searched PubMed, EMBASE, and ISI databases for relevant studies through 10 May 2015. Prospective studies reporting the risk of SUA elevation on the incidence of MetS/NAFLD were enrolled. Pooled HR of MetS was 1.55 (95%CI: 1.40–1.70) for the highest versus lowest SUA categories, and 1.05 (95%CI: 1.04–1.07) per incremental increased in SUA of 1 mg/dl. The pooled HR of MetS in younger women was higher than age-matched men and older women (1.17 vs. 1.05 and 1.04, respectively, *P *< 0.05). Individuals in the highest SUA category had a 40% greater risk of disease NAFLD occurrence. Dose-response increment of NAFLD events was 1.03 (95%CI: 1.02–1.05). A positive relationship with a linear trend for SUA elevation with MetS and NAFLD in different genders was examined by a dose-response meta-analysis (*P *< 0.001).SUA assay is useful in screening metabolic disorders for linear trend between its elevation and MetS/NAFLD incidence. SUA-lowering therapy is a potential strategy for preventing systemic/hepatic metabolic abnormalities.

At least one-fourth of the global adult population suffers the health burden of the metabolic syndrome (MetS)^1^,^2^. MetS is a complex collection of clinical manifestations, including abdominal obesity, dyslipidemia, hyperglycemia, and hypertension^3^,^4^. More than only a benign disease process, MetS increases the risk of cardiovascular disease (CVD) and type 2 diabetes mellitus (T2DM) events by approximately 2- and 6-fold, respectively^5^,^6^. MetS is considered to be a crucial mediator from simple over-nutrition to severe body lesion by promoting inflammation^7^,^8^, inducing an approximate 40% higher mortality in adults^6^.

As a result of insulin resistance, non-alcoholic fatty liver disease (NAFLD) represents excessive adipocyte accumulation in the liver^9^, and is considered to be a metabolic disorder manifested in liver^10^. NAFLD is the most common cause of liver function abnormality^11^, affecting >20% of the global population^12^. As a cause and consequence of MetS^13^, NAFLD is also linked to a higher incidence of T2DM and CVD, and a higher mortality rate^14^,^15^.

Serum uric acid (SUA) is the end-product of purine nucleotide catabolism. Hyperuricemia is associated with UA deposition in joints and tissues. In addition to contributing to the pathogenesis of gout, arthritis, and chronic nephropathy, hyperuricemia is associated with so-called “cardio-metabolic diseases” including CVD, T2DM, and MetS^16^,^17^. Choi *et al.* reported a significantly higher prevalence (up to 60%) of MetS in the hyperuricemic population^18^. Hyperuricemia might be an indicator for early diagnosis and prevention of MetS^19^. Thus, SUA maintenance in an appropriate range might relieve the health burden from MetS^20^.

Hyperuricemia was also independently associated with the NAFLD development, even after adjusting for potential confounders including MetS features and insulin resistance^21^,^22^,^23^. Regarding the potential role, hyperuricemia exerts in promoting pro-inflammatory, pro-oxidant function, and insulin resistance in adipose tissue^24^,^25^,^26^. Hyperuricemia might induce more oxidative stress within the liver^27^, and activate the “second-hit” process underlying the NAFLD pathogenesis^28^. Recent studies have shown a close correlation between the SUA level and histologic severity of NAFLD-related liver damage^29^,^30^.

Given the close association between UA and MetS/NAFLD, some prospective studies have explored the role of SUA in predicting MetS or NAFLD^19^,^31^,^32^,^33^,^34^,^35^,^36^,^37^,^38^,^39^,^40^,^41^,^42^; however, inconsistent and controversial results indicated that some potential confounders might influence the predictive role of SUA in monitoring MetS and NAFLD. Therefore, we performed a systematic review and meta-analysis to quantitatively evaluate the risk trend of the MetS/NAFLD incidence followed by SUA variation based on published papers. Latent confounders were searched by subgroup analysis. This is the first evidence-based study to evaluate the risk of hyperuricemia-related systemic/hepatic metabolic disorders in general populations. The current study is intended to clarify the relationship between hyperuricemia and metabolic disorders, and provide potential approaches for prevention of MetS, NAFLD, and related complications.

## Materials and Methods

### Search strategy

We conducted this systematic review and meta-analysis according to the Preferred Reporting Items for Systematic Reviews and Meta-analyses (PRISMA) guideline (see checklist S1 and flow diagram S1)^43^. A medical librarian, with rich experience in systematic reviews, participated in the search strategy design process. A relevant literature search (without language restrictions) was conducted using the following databases: PubMed; Embase; and the Institute for Scientific Information (ISI) database (last updated: 10 May 2015). The following medical subject headings were used for searching the relevant literatures, with research subjects restricted to humans: “uric acid;” “urate;” “gout;” “UA;” “metabolic syndrome;” “syndrome X;” “insulin resistance syndrome;” “MetS;” “non-alcoholic fatty liver disease;” “non-alcoholic steato-hepatitis;” “steatosis;” “NAFLD;” and “NASH.” Additional manual search was performed if relevant papers were omitted. The search strategy for the database is available in Table S1.

### Eligibility criteria

Articles were included for analysis based on the following criteria: 1) prospective cohort studies performed in adults published as original data; 2) SUA was provided and the group with the lowest SUA was designated as the reference; 3) all of the enrolled participants were free of observational end points (MetS or NAFLD) at baseline; 4) the continuous or dichotomous risk indicator of MetS/NAFLD incidence caused by SUA elevation was provided or could be obtained by calculation; 5) the diagnostic criteria of end points were clearly defined. Literature reviews, retrospective or cross-sectional studies, case-control studies, adolescents studies were excluded.

### Validity assessment

The quality of the primary studies was independently evaluated by two authors (ZTL and SPQ) using the Newcastle-Ottawa Scale (NOS) as an assessment for non-randomized studies^44^. The NOS consisted of three major components, including participants selection (four stars), inter-study comparability (three stars), and outcome assessment (two stars; Table S2). Studies with more than six stars were considered to be high quality.

### Data extraction

Two authors (ZTL and SPQ) independently extracted data from original reports using a standardized form with pre-defined criteria. Agreement was measured by Cohen’s kappa^45^. The risks with the most comprehensive covariates adjusted were extracted to avoid potential bias. The study characteristics were extracted as follows: first author; publication year; country of data origin; participants characteristics (including total number, number of cases, gender, and age distribution); comparisons, calculation method; follow-up duration; definition of disease outcome (hyperuricemia, MetS, or NAFLD); risk of disease as a function of SUA variation; and adjusted covariates.

### Rescaling of exposure

For consistency, the SUA value was presented as mmol/L divided by 0.0595 to convert to mg/dl. When studies only reported SUA range, the mid-point was defined as the average of the lower and upper boundaries in each category. When the highest category was open-ended, the median value was assigned as the lower end value multiplied by 1.2^46^.

### Statistical analysis

We selected the HR (OR was combined after converting into risk ratio [RR] by previous method^47^) and 95% confidence interval (CI) to assess the SUA-MetS/NAFLD association. Age- and gender-specific HR was presented separately if provided in the literature.

First, we compared the pooled HR of the end point (MetS/NAFLD) incidence between the highest and lowest SUA categories. Second, the linear dose-response risk of the MetS/NAFLD incidence for each 1 mg/dl increment in SUA elevation was estimated using a generalized least squares (GLST) calculation based on reported data (SUA concentration of the median dose, total/case number, and corresponding effects compared to the lowest SUA subgroup)^46^,^48^. Otherwise, a two-stage meta-analysis was performed to determine the non-linear dose-response relationship between the SUA level and MetS/NAFLD incidence. The restricted cubic spline was used to model the SUA level at fixed knots (including 5%, 35%, 65%, and 95% of the distribution)^49^,^50^. The generalized least-squares and multivariate maximum likelihood methods were utilized to estimate a summary non-linear dose-response relationship, taking random effects into consideration^51^. A *P*-value for non-linearity was calculated using a null hypothesis test and the coefficient of the second spline was equal to 0.

Furthermore, subgroup analysis was also performed to identify the effect of potential confounders. A cumulative meta-analysis was performed to investigate the changes over time in the pooled estimate of effect size. Sensitivity analysis was conducted to investigate the potential influence of a single study on the results.

Statistical heterogeneity was evaluated using the chi-square-based Q test and I^2^ test. We defined low, moderate, and high heterogeneity as 25%, 50%, and 75% for I^2^, respectively^52^. A fixed-effect model was performed if no significant heterogeneity was observed (*P*-value > 0.05 and I^2 ^< 50%). Otherwise, a random-effect model was used^53^. Begg and Egger’s test^54^,^55^ were used to estimate potential publication bias. A *P *< 0.05 for was considered as significance. All of the statistical analyses were performed using Stata 12.0 version software (Stata Corp, College Station, TX, USA).

## Results

### Literature retrieval

**Figure 1:**
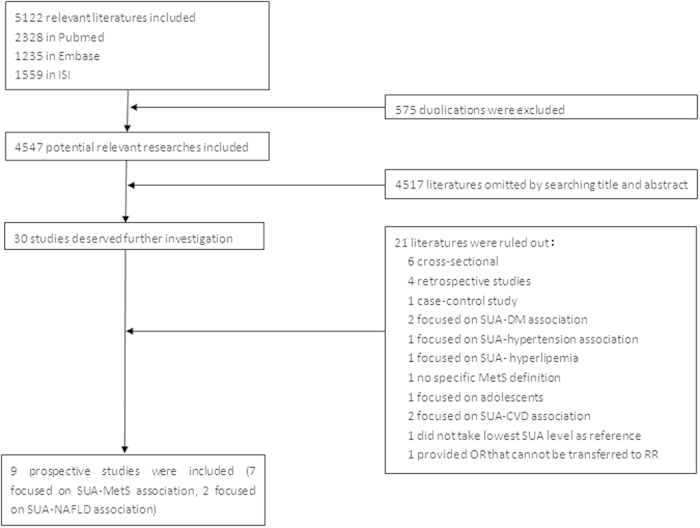
Flow diagram of eligible literature selection.

We screened 4,547 potentially relevant articles, after excluding 575 duplicates in 3 databases (PubMed, Embase, and ISI). The final enrolled number of eligible articles was 9 (7 and 2 on the UA-MetS and UA-NAFLD association, respectively) with high concordance between reviewers (Cohen’s kappa = 0.787). The flow chart for literature selection was shown in Fig. 1.

### Quality assessment, study characteristics, and bias assessment

According to the NOS assessment system, all of the enrolled studies were considered to be high quality. Studies varied in NOS score from 7 to 9 (average score = 7.67). The details of quality assessment are shown in Table S3.

The study characteristics are shown in Table 1. Seven studies^19^,^31^,^32^,^34^,^35^,^38^,^40^,, including 23081 men, and 12195 women were focused on evaluating the risk of MetS incidence, and 2 studies^33^,^42^, including 4492 men, and 8139 women were focused on the NAFLD risk, as a function of SUA variation. Six studies reported the effects of UA on MetS/NAFLD separated by gender, one study reported result in the population combining males and females, and two studies only reported the results on males. Seven, one, and one study were conducted in East Asia, USA, and Europe, respectively, with follow-up durations ranging from 2.5–5.5 years, and sample sizes from >1000 to >9000 subjects. The age of participants ranged from 20–65 years. The cut-off value of hyperuricemia was defined as >7 mg/dl for males, and >6 mg/dl for females. According to self-defined cut-off values, the prevalence of hyperuricemia ranged from 11.9%–33.8% in males, and 4.7–25.2% in females at baseline. Two studies defined MetS by Joint Interim criteria^56^ and two studies used the American Heart Association/National Heart, Lung, and Blood Institute (AHA/NHLBI) criteria^57^. Alternatively, the International Diabetes Federation (IDF), modified National Cholesterol Education Program Adult Treatment Panel III (NCEP-ATP III), and Japanese criteria^58^,^59^,^60^ were used for the enrolled studies (Table S4). The MetS incidence in enrolled studies ranged from 11%–36% in males, which was always higher than corresponding data in females (3.5%–20%) from the same cohort. The NAFLD incidence ranged from 11.8%–29.9% as a function of follow-up duration in East Asia. Cox proportional hazards, logistic, and Poisson regression models were utilized for calculating HR, OR, and IRR in six, two, and one study, respectively. Age, gender and MetS components were adjusted as key covariates in all studies. Six studies reported dichotomous risk (high *vs.* low SUA category) and three studies reported the dichotomous and continuous risk. The trend in MetS/NAFLD incidence followed by SUA variation was presented with great deviation on risk extent (Figure S1). Two studies^35^,^40^ reported the continuous effects on incidence of individual MetS components followed per standard deviation (SD) of SUA elevation.

### SUA and MetS incidence

#### High versus low

**Figure 2:**
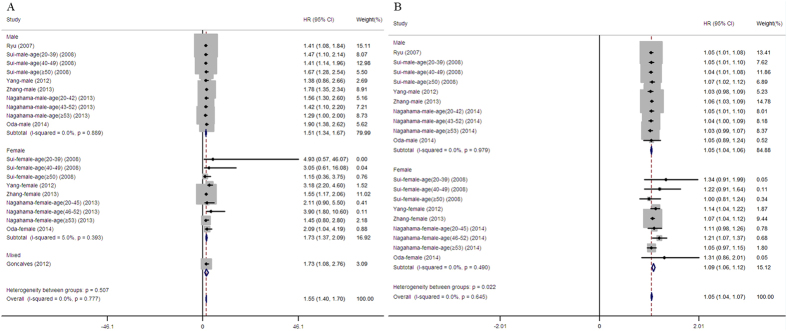
Forest plot of association between serum uric acid and metabolic syndrome in prospective studies. (**A**) Pooled hazard ratios of metabolic syndrome compared between highest and lowest serum uric acid categories; (**B**) Pooled hazard ratios of metabolic syndrome followed per 1 mg/dL of serum uric acid elevation.

The pooled HR for incident MetS based on a comparison of subjects in the highest category with subjects in the lowest category in 7 studies was 1.55 (95% CI: 1.40–1.70) with low heterogeneity (I^2 ^= 0%, P = 0.777; Fig. 2A). No significant difference regarding the SUA-MetS association was observed between males and females (*P *> 0.05).

#### Dose-response analysis

The continuous HR of the MetS incidence per 1 mg/dl increment in SUA elevation was provided as original data or extracted by calculation in 6 studies (Fig. 2B). An approximate 5% increase in MetS occurred per 1 mg/dl increment in SUA elevation as a function of follow-up duration with low heterogeneity (I^2 ^= 0% , P = 0.645). The risk for MetS was higher in females (1.09, 95% CI: 1.06–1.12) than males (1.05, 95% CI: 1.04–1.06) significantly (*P *= 0.022).

A comparison of individual MetS features, including obesity, hypertriglyceridemia, hyperglycemia, hypertension, and low HDL-C incidence as a function of SUA elevation was performed in two studies (Figure S2). The SUA elevation was associated with hypertriglyceridemia (1.41, 95%CI: 1.26–1.57), central obesity (1.27, 95% CI: 1.11–1.46), and hypertension (1.30, 95%: 1.15–1.45).

**Figure 3:**
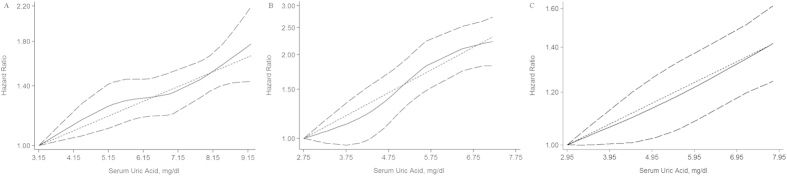
Dose-response relations between serum uric acid levels and risk of metabolic syndrome/non-alcoholic fatty liver disease in prospective studies. (**A**) Restricted cubic splines and generalized least squares dose-response models on evaluation of association between uric acid and risk of metabolic syndrome in men; (**B**) Restricted cubic splines and generalized least squares dose-response models on evaluation of association between uric acid and risk of metabolic syndrome in women; (**C**) Restricted cubic splines and generalized least squares dose-response models on evaluation of association between uric acid and risk of non-alcoholic fatty liver disease. The solid line represents the fitted hazard ratios curve compared to the subgroup with the lowest mean dose of uric acid, and flanked dotted line is 95%CI of this risk by restricted cubic splines model. Middle dotted line represents the weighted regression index compared to subgroup with lowest mean dose of uric acid by generalized least squares model.

The dose-response relationships between the baseline SUA level and MetS risk were demonstrated after pooling gender-specific results by different models. An insignificant non-linear relationship existed between SUA variation and the incidence of MetS in both genders (*P *= 0.3957 for males and *P *= 0.5014 for females; Fig. 3A,B). Convincing evidence revealed that a linear model was better fit to illustrate the SUA-MetS association (*P* for linear trend < 0.001 for both genders).

#### Subgroup, sensitivity analysis, and cumulative meta-analysis

**Figure 4:**
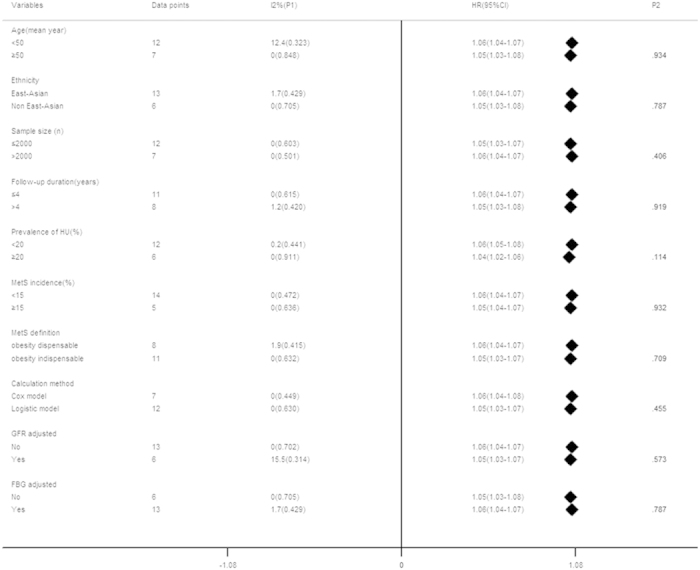
Subgroup analysis of factors influencing the dose-response risk of metabolic syndrome associated with uric acid elevation. *P-value was calculated by metan between subgroups.

**Figure 5:**
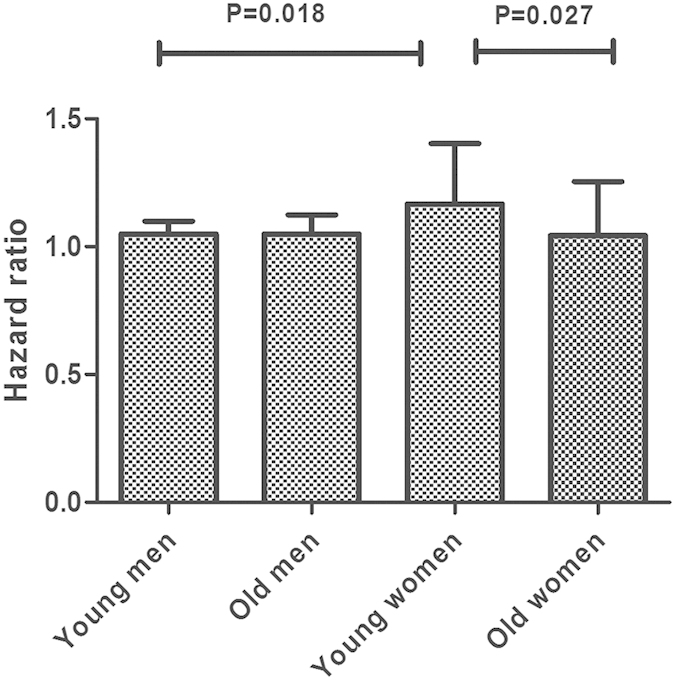
Comparison of dose-response risk of metabolic syndrome between age-confined subgroups. Young men/women represents the first two age tertiles of subjects in enrolled studies, old men/women represents the third tertile of subjects in enrolled studies. P1 represented the heterogeneity within subgroups, P2 represented the heterogeneity between subgroups. P value was calculated between subgroups based on metan calculation.

Subgroup analyses of the summarized dose-response effect on the UA-MetS association are shown in Fig. 4. Stratified analyses were classified by mean age, ethnicity, sample size, follow-up duration, baseline prevalence of hyperuricemia, calculation method, MetS definition and incidence, adjustment of glomerular filtration rate (GFR) or fasting blood glucose (FBG). There was no significant difference between subgroups classified by any potential confounders (*P *> 0.05 for inter-subgroup comparison). Further, to identify the potential interaction between age and gender on the UA-MetS association^61^, a subgroup comparison was conducted in two studies^32^,^38^ reporting gender-specific HRs in a clearly defined age range. The pooled HR in younger females (a combination of the youngest and middle age tertiles) was 1.17 (95% CI: 1.07–1.26), prominently higher than age-matched males (*P *= 0.018) and older females (*P *= 0.027; Fig. 5).

The trend of the dose-response effect was also evaluated in studies excluding the data from subjects with the highest open-ended UA levels. After exclusion, the upper limit range of UA was 6.5–7.7 mg/dl in males and 4.6–6 mg/dl in females, which was lower than the commonly-defined normouricemia threshold (7.2 mg/dl for males, and 6.0 mg/dl for females)^62^ in most studies (except a slightly higher male boundary value [7.7 mg/dl] in one study^34^). As shown in Figure S3A, approximate 4% increase for males and 8% increase for females on pooled HR in subjects mainly within normal SUA range.

There was no single study that significantly altered the pooled dose-response results. After omitting one study and re-evaluating summarized HRs of the remaining studies in turn, the range of the estimated effect did not exceed 0.5% (1.042–1.070; Figure S4). A cumulative meta-analysis showed constant pooled estimates of effect size over time (Figure S5).

#### Publication bias analysis

Visual inspection of the Begg funnel plot, by the SE of the log HR from each study was plotted against the log HR (Figure S6). Although slight asymmetry was observed in the Begg funnel plot, no publication bias was detected with the Begg’s test (*P *= 0.504) and Egger’s test (*P *= 0.105).

### SUA and NAFLD incidence

#### High versus low

**Figure 6:**
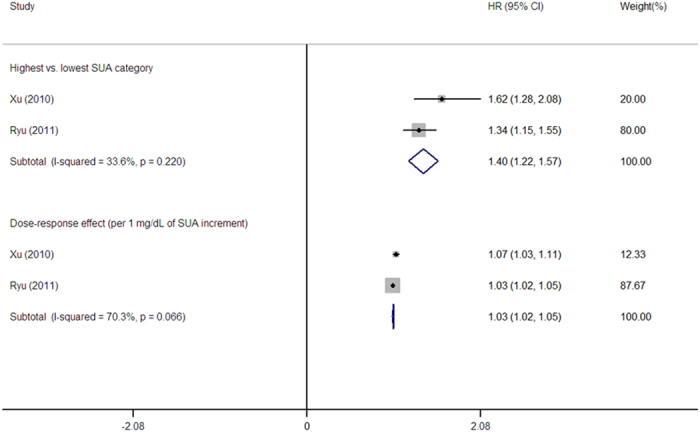
Forest plot of association between serum uric acid and non-alcoholic fatty liver disease incidence in prospective studies.

Two studies reported a risk for NAFLD associated with SUA elevation (Fig. 6). The pooled HR between the highest versus lowest SUA category was 1.40 (95% CI: 1.22–1.57) with lower-to-moderate heterogeneity (I^2 ^= 33.6%, *P *= 0.220).

#### Dose-response meta-analyses

The summary RR for the NAFLD incidence as a function of 1 mg/dl SUA increment was 1.03 (95% CI: 1.02–1.05), with a moderate-high degree of heterogeneity (I^2 ^= 70.3%, P = 0.066; Fig. 6). The dose-response SUA-NAFLD association was also examined using the cubic spline model (Fig. 3C). We did not detect a significant non-linear dose-response relationship after pooling the results (*P *= 0.9232). A weighted linear relationship was demonstrated (*P *< 0.001). Of note, both studies adjusted all the indicators of MetS components, including body mass index (BMI), triglycerides, HDL-C, FBG, blood pressure (BP), alcohol intake, and smoking status for enrolled subjects (Table 1).

After excluding the subjects in the highest SUA categories, the SUA level of the enrolled participants was confined in 6.89 mg/dl for males, and 5.03 mg/dl for females, which was lower than the previously defined cut-off for hyperuricemia^62^. The re-summarized dose-responded HR was 1.03 (95% CI: 1.01–1.06), presenting moderate-to-high heterogeneity (I^2 ^= 66.9, *P *= 0.082; Figure S3B).

Significant heterogeneity, especially with respect to dose-response effect of the NAFLD outcome, might be due to the difference in gender distribution (one study included both genders, while another study only enrolled males; Table 1). However, few studies precluded any meaningful subgroup, sensitivity, or publication bias analyses.

### Risk differences on MetS and NAFLD incidence associated with SUA variation

According to pervious statistics for disease incident rates^63^, the estimated risk for MetS was 401.8 and 238.8 cases per 100 000 individuals per year as a function of 1 mg/dl SUA increment in males and females. The corresponding estimates for NAFLD incidence per 1 mg/dl increment in SUA levels was 142.5 cases per 100,000 individuals per year.

## Discussion

Based on a meta-analysis of prospective studies, a significant association was demonstrated between SUA levels and the risk for systemic/hepatic metabolic disorders. We synthesized the dose-response results from six studies, including 34222 participants and 5032 cases of MetS outcomes as a function of follow-up duration. Approximate 5% increment for males and 9% increment for females were observed on the MetS incidence per 1 mg/dl of SUA elevation. Based on data from two prospective studies, the dose-response risk of NAFLD per 1 mg/dl increase on SUA was approximately 1.03. Subgroup analyses indicated that younger females (<52 years) was more sensitive to develop MetS on the same SUA elevation degree than age-matched males and older females (>52 years). We speculated that there was a linear relationship between SUA elevation and MetS/NAFLD incidence.

It has long been thought that there is a causal role and predictive value for hyperuricemia with respect to MetS development due to the promotion of endothelial dysfunction, inflammation, and reactive oxidative stress (ROS)^20^,^37^,^64^; however, the extent of this effect is still uncertain due to the complex interrelationship^65^. After pooling previous studies, we showed that a SUA elevation was a stable and continuous risk factor for MetS events (Fig. 2). Consistent with previous results from retrospective cohort and cross-sectional studies^66^,^67^,^68^, subgroup analysis revealed that this association was prominent even in subjects with normouricemia (Figure S3). Recently, a J-shaped association between the SUA level and MetS events drawn from Taiwanese elderly males (>60 years), indicated that hypouricemia (defined as a SUA < 4.5 mg/dl) does not prevent MetS^39^. In our meta-analysis, no enrolled study has ever reported the risk of SUA-related MetS incidence separately in elderly males with hypouricemia. Therefore, the results should be explained with caution when extrapolated to older populations.

Concerns about the menopausal status-specific effects on the association between SUA level and MetS or its individual components have been raised in recent cross-sectional studies with inconsistent results^67^,^69^,^70^,^71^. Some scholars have attributed the stronger influence of hyperuricemia on MetS occurrence due to the uricosuric effects of estrogen^65^,^72^. The benefit of hormone replacement therapy reducing the risk of hyperuricemia and related complications also confirmed this speculation^73^,^74^. Our meta-analysis showed a three-fold higher risk of MetS outcome in younger female participants (<52 years, thus excluding the majority of postmenopausal women^75^), than males and older females (Fig. 4), indicating that estrogen, rather than gender, might be a crucial determinant interfering with the SUA-MetS association.

Previous meta-analyses have summarized the positive association between the SUA level with hypertension and diabetes incidence, as individual MetS components^76^,^77^; however, no study has ever compared the differential effects of hyperuricemia on individual MetS features. After pooling the reported data, a significant association was observed between SUA levels and the incidence of most individual MetS components including hypertriglyceridemia, hypertension, adiposity, and low HDL-C, except for hyperglycemia (Figure S2). SUA-MetS association was independent of the relationship between SUA and DM (Fig. 4). Interestingly, this weak correlation between SUA and hyperglycemia was also observed in previous cross-sectional studies^21^,^78^,^79^, indicating that the influence of SUA might be inherently deviated from mediating the FBG level in MetS as a disease entity. Taniguchi *et al.* speculated the effect of hyperuricemia on insulin resistance was dependent on obesity and ethanol intake^80^; however, the exact underlying mechanism is unclear.

NAFLD is considered to be a metabolic disorder specifically manifested in the liver^81^,^82^. Compared to MetS, few studies have focused on the risk for NAFLD as a function of SUA variation longitudinally and mainly based on East Asia^33^,^42^. Consistent with previous studies^22^, a 3% linear increase in the incidence of NAFLD with 1 mg/dl increase on SUA was observed whether or not within the normal range of SUA, and independent of MetS and lifestyle factors. The HR was higher for the combination of genders than males, indicating that gender might interfere with the pooled results (Fig. 6). The discrepant results might be due to a potential protective effect of estrogen against hepatic steatosis^83^. Previous studies have reported that hyperuricemia is associated with severe histologic hepatic damage, and poorer long-term survival in NAFLD patients^29^,^84^. Further, age- gender-, and ethnicity-specific longitudinal studies with additional information are warranted to fully disclose the SUA-NAFLD relationship.

**Figure 7:**
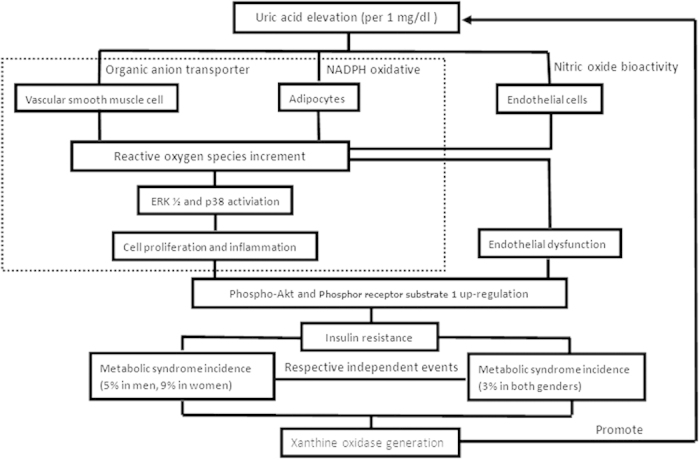
Potential mechanisms between serum uric acid elevation and incident metabolic disorders.

The role of SUA on the pathogenesis of systemic and hepatic metabolic abnormalities (causal or only consequent) is a matter of debate^65^. By reducing the nitric oxide bioavailability, uric acid stimulates intracellular oxidative stress and impairs endothelial function^20^,^85^,^86^,^87^. Uric acid also has direct pro-inflammatory and pro-oxidative effects on adipocytes^25^; these effects play critical roles in the development of insulin resistance^88^,^89^. MetS and NAFLD might occur via the regulation of hyperuricemia on insulin resistance. In addition, recent experimental studies have shown a direct effect of hyperuricemia on insulin resistance by increasing ROS release and inhibiting the insulin signaling pathway, thus supporting previous speculation^90^. A causal link between SUA elevation and MetS/NAFLD incidence was observed after pooling the enrolled prospective data via a meta-analysis. Of note, an insignificant SUA-MetS association existed in study^41^ adjusted for HOMA-IR^91^, suggesting dependence of this pathogenesis on differentiating insulin resistant status caused by uric acid. Further studies are needed to confirm this viewpoint. Otherwise, SUA elevation was also a concomitant phenomenon followed with the xanthine oxidase generation in large amount caused by some metabolic disorders. SUA had anti-oxdative effect, and its compensatory elevation indirectly reflected the disease status in oxidative stress^65^,^92^. Allopurinol (xanthine oxidase inhibitor) was speculated to play its role on reducing oxidants, rather than direct hypouricemic effect^20^. Combined with our data, the potential mechanism underlying SUA-MetS/NAFLD association was summarized and quantitatively presented in Fig. 7.

The strength and availability of our results should be mentioned. For the approximate linear dose-response relationship, SUA might be an available biomarker in predicting and screening the incidence of metabolic disorders with high cost-effectiveness. Uric acid reduction might be a future therapeutic target for preventing MetS or NAFLD^93^ as a common risk factor for severe disease, such as CVD^94^, due to the potential causal effect of hyperuricemia on metabolic abnormalities. And this theory was also proved by a previous animal study in rats^20^. Our results comprehensively evaluated the risk for MetS/NAFLD as a function of SUA elevation on the natural course without intervention by drug therapy in various populations, which might help for better evaluating drug efficiency, by individualized adjustment of inherent tendency of hyperuricemia on metabolic disease. We also calculated the precise MetS sufferers, and showed a higher morbidity rate for MetS (cases per 100,000 individuals per year) at baseline in the lowest SUA category amongst males compared to females (1627.8 *vs.* 742.4 [data not on shown]). A similar dose-response HR might mean more patients in a given male population. It cannot be neglected in spite of relatively lower pooled HRs in males.

Limitations of this meta-analysis should be mentioned. Prospective studies focusing on the SUA-NAFLD association were mainly conducted in East Asia, and this relationship should be validated in subsequent studies involving other ethnicities. The upper limit cut-offs for SUA levels were not defined with a unified range. Distinct definition might cause slight deviation on the SUA intervals when comparison was performed in subjects with exclusion of highest SUA categories (Figure S3), but majority of them were under the recognized cut-offs for normal SUA values^62^. We considered results in figure S3 can illustrate the prominent UA-MetS/NAFLD association in population even within relatively low SUA. Inconsistence on risk measures and statistical methods used across enrolled studies as disadvantages for combination of enrolled results should be mentioned. HR and OR from different statistic models had discrepant meaning and potential heterogeneities might exist when combining these different indicators. So, OR was transferred to RR^47^ before data combination, for increasing inter-study comparability. The SUA level fluctuates for multi-covariates including purine-rich foods (meat and beer), different ethnicities, genetic background, and chronic kidney disease (CKD) status^95^,^96^,^97^. However, adjustment of the GFR (as an indicator of CKD), and ethnicity caused little impact on final results (Fig. 4). Enrolled studies adopted different criteria for the diagnosis of MetS (Table S4), which might cause bias on defining patients and non-patients. Taking central obesity as dispensable or indispensable covariates in MetS definition did not have prominent deviation on pooled HRs (Fig. 4), indicating a slight effect on the overall results. SUA might selectively influence the occurrence of MetS with a specific etiology^20^, and we cannot clarify the specific disease cause in enrolled subjects.

In conclusion, a consistent and linear causality from uric acid increase on MetS/NAFLD incidence was observed through meta-analysis of prospective studies. SUA might be an individualized predictor in screening incidents of systemic/hepatic metabolic abnormities. Lowering the SUA level might be a potential treatment for preventing comprehensive metabolic disorders. Well-designed randomized controlled trials of high quality are needed to confirm these effects.

## Additional Information

**How to cite this article**: Liu, Z. *et al.* Dose-response Relationship of Serum Uric Acid with Metabolic Syndrome and Non-alcoholic Fatty Liver Disease Incidence: A Meta-analysis of Prospective Studies. *Sci. Rep.* **5**, 14325; doi: 10.1038/srep14325 (2015).

## Supplementary Material

Supplementary Information

## Figures and Tables

**Table 1:** Characteristics of the ten cohort prospective studies included in meta-analysis

First author, publication year [ref]	Country	Enrolled study population (case^a^/total, baseline characteristics)	Gender (female /male)	Age (range, mean ± SD)	Hyper- uricemia (definition [mg/dl], prevalence [%])	Comparison (SUA, mg/dl)	Follow-up (years, mean ± SD)	Outcome	HR (95%CI)	Calculation method	Adjusted covariates
Ryu *et al.* 2007[31]	Korea	708/4779 without MetS, without medication, and without malignancy	0/4779	(30–39) 33.5 ± 2.5	NG	Highest quintile *vs*. lowest quintile (≥6.5 vs. <5.5)	3	MetS	1.41 (1.08–1.84)	Cox proportional hazards model	Age, GGT, FBG, BMI, HDL-C, TG, BP
Sui *et al.* 2008[32]	USA	M: 1120/8429 without MetS, without CVD, without cancer, with normal cardiogram	1260/8429	M: HU(-): 43.6±9.2; HU(+): 43.5 ± 9.0	M: >7, 18.9	M: Highest tertile *vs*. lowest tertile (≥6.71 vs. <4.97)	5.5 ± 4.7	MetS	M(20–39yr): 1.54(1.10–2.14) M(40–49yr): 1.50(1.14–1.96) M(≥50yr): 1.80(1.28–2.54)	Multivariable logistic regression model	Age, examination year, BMI, current smoking, alcohol intake, number of baseline metabolic risk factors, family history of disease, and treadmill test
		F: 44/1260 without MetS, without CVD, without cancer, with normal electro- cardiogram		F: HU(−): 44.2 ± 9.3; HU(+): 44.1 ± 9.2	F: >6, 4.7	F: Highest tertile *vs*. lowest tertile (≥4.6 vs. <3.8)			F(20–39yr): 5.12(0.57–46.07) F(40–49yr): 3.14(0.61–16.08) F(≥50yr): 1.16(0.36–3.75)		duration
Yang*et al.* 2012[34]	Chinese Taiwan	M: 214/1748 without MetS	2109/1748	M: T1^b^: 44.44 ± 16.14; T2:38.85 ± 16.52; T3:39.61 ± 16.80	M: ≥7.7, 33.8	M: Highest tertile *vs*. lowest tertile (≥7.7 vs. <6.4)	5.41 ± 0.36	MetS	M: 1.38 (0.86–2.66)	Cox proportional hazards model	Age, variations of BP, TG, HDL-C, FBG, and WC
		F: 262/2109 without MetS		F: T1:39.32 ± 13.67; T2:39.75 ± 15.13; T3:42.90 ± 14.63	F: ≥6.6, 18.6	F: Highest tertile *vs*. lowest tertile (≥5.8 vs. <4.7)			F: 3.18 (2.2–4.6)		
Goncalves *et al.* 2012[35]	Portugal	F: 237/1054 without MetS	639/418	49.6 ± 14.7	M: >7, F: > 6 17.6	HU(+)/HU(−) (≥7 *vs.*<7 for men, ≥6 *vs.*<6 for women)	5±3.33	MetS	1.73(1.08–2.76)	Poisson regression model	Age, sex, and education, smoking, alcohol intake, protein, calories consumption, and total physical activity, one or
						Per SD increase of UA level vs. before			1.22(1.05–1.42)		two features of MetS at baseline
Zhang *et al.* 2013[19]	China	M:776/2181 without MetS	4442/2957	M: 51.1 ± 14.6	M: >7,11.9	M: HU(+)*vs*. lowest quartile (>7 vs. <5.3)	3	MetS	M: 1.78 (1.35–2.34)	Cox proportional hazards model	Age, BMI, smoking status, drinking status, habit of regular exercise, BP, LDL-C, TG, HDL-C and FBG
		F:749/3693 without MetS		F: 46.1 ± 14.0	F: >6, 12.6	F: HU(+) *vs*. lowest quartile (>6 vs. <4.1)			F: 1.55 (1.17–2.06)		
Nagahama *et al.* 2013[38]	Japan	M(T1):264/1056 without MetS	2792/3144	MT1:(20–42)	M(T1): ≥7,32.0	M:HU(+)/HU(-)	4	MetS	M(T1): 1.8(1.3–2.6)	Multivariable logistic	Alcohol consumption, smoking status, WC,BP,
		M(T2):269/784 without MetS		MT2: (43–52)	M(T2): ≥7,31.0	(≥7/<7)			M(T2): 1.6(1.1–2.2)	regression model	dyslipidemia, FBG,GFR and medication use for
		M(T3):246/1035 without MetS		MT3: (53–65)	M(T3): ≥7,25.4				M(T3): 1.4(1.0–2.0)		hypertension, dyslipidemia, diabetes
		F(T1):40/942 without MetS		FT1: (20–45)	F(T1): ≥6,5.9	F: HU(+)/HU(−)			F(T1): 2.2(0.9–5.5)		
		F(T2):44/910 without MetS		FT2: (46–53)	F(T2): ≥6,8.7	(≥6/<6)			F(T2): 4.4(1.8–10.6)		
		F(T3):81/940 without MetS		FT3: ≥ 54	F(T3): ≥ 6,15.0				F(T3): 1.5(0.8–2.8)		
Oda *et al.* 2014[40]	Japan	M: 177/1606 without MetS	953/1606	M: 51.5 ± 9.6	M: ≥7,23.8	HU(+) *vs*. lowest quantile (≥7 vs.1.1–5.2)	2.5	MetS	2.615 (1.918–3.566)	Cox proportional hazards models	Age, smoking, drinking, physical activity, medication for hypertension, hyperlipidemia,
						Per 1 SD increase of UA level vs. before			1.282 (1.097–1.499)		and diabetes, histories of CHD and stroke, MetS components
						Per 1 increase of UA level vs. before			1.052 (0.895–1.236)		
		F: 71/953 withoutMetS		F: 51.0 ± 9.7	F: ≥6,25.2	HU(+) *vs*. lowest quantile (≥6 vs.1.8–3.7)			2.088 (1.04–4.19)		
						Per 1 SD increase of UA level vs. before			1.354 (1.041–1.762)		
						Per 1 increase of UA level vs. before			1.313 (0.857–2.013)		
Xu *et al.* 2010[42]	China	813/6890 without NAFLD, alcohol abusers, hepatotoxic drugs medication, and hepatitis)	4492/2398	44.4 ± 12.7	M: ≥7.0 F: ≥6.0	Highest quintile *vs*. lowest quintile (M: ≥6.89 vs.<4.96, F: ≥ 5.03 vs. <3.45)	3	NAFLD	1.62 (1.26–2.08)	Cox proportional hazards models	Age, gender, alcohol intake, BMI, waist circumference, BP, ALT, AST, GGT, TG, total cholesterol, HDL-C, LDL-C, FPG, creatinine and BUN
Ryu *et al.* 2011[33]	Korea	1717/5741 without NAFLD, alcohol abusers, ALT elevation, liver disease, medication,	0/5741	36.7 ± 4.9	≥7.0, 14.1%	Highest quartile *vs*. lowest quartile (6.5–11.5 vs.0.8–5.1)	4.9	NAFLD	1.34 (1.15–1.55)	Cox proportional hazards models	Age, BMI, smoking, alcohol intake, exercise, total cholesterol, HDL-C, TG, FPG, BP, insulin, hsCRP, and the MetS presence
		malignancy, CVD and diabetes				HU(+) *vs.* HU(−) (≥7 *vs.* <7)			1.21 (1.07–1.38)		
						Per 1 increase of UA level vs. before			1.11(1.06–1.16)		

^a^represented the number of target disease occurrence in prospective studies.

^b^represented the age in subgroups classified by uric acid tertiles.
