# Supplementary material for: Dose-response Relationship of Serum Uric Acid with Metabolic Syndrome and Non-alcoholic Fatty Liver Disease Incidence: A Meta-analysis of Prospective Studies
Source: Sci Rep. 2015 Sep 23;5:14325. doi: 10.1038/srep14325 (PMC4585787; doi:10.1038/srep14325)
Supplement: Supplementary Information [file srep14325-s1.pdf]

Dose-response Relationship of Serum Uric Acid with Metabolic Syndrome and Non-alcoholic Fatty Liver Disease Incidence: AMeta-analysis of Prospective Studies

Zhengtao Liu[1,2,3*], Shuping Que[4*], Lin Zhou[1,2,3]

Author affiliation:

1. Key Laboratory of Combined Multi-Organ Transplantation, Ministry of Public Health and Key Laboratory of Organ Transplantation of Zhejiang Province, Hangzhou, 310003, Zhejiang province, China.

2. Collaborative Innovation Center for Diagnosis and Treatment of Infectious Diseases, Hangzhou, 310003, China.

3. Division of Hepatobiliary and Pancreatic Surgery, Department of Surgery, First Affiliated Hospital, School of Medicine, Zhejiang University, Hangzhou, 310003, China.

4. Department of Pediatrics, Women and children's hospital of Guangxi, Nanning, 530005, Guangxi province, China

*Correspondence to Zhengtao Liu, Key Laboratory of Combined Multi-Organ Transplantation, Ministry of Public Health and Key Laboratory of Organ Transplantation of Zhejiang Province, 79# Qingchun road, Shangcheng district, Hangzhou, 310003, Zhejiang province, China.

E-mail: liuzhengtaocn@hotmail.com

Tel: +86-571-87236466, Fax: +86-571-87236466

Conflict of interest: The authors declare no conflict of interest.

*These authors contributed to the work equally

Figure S1 Risk measures of metabolic syndrome/non-alcoholic fatty liver disease according to study specific mean dose of serum uric acid

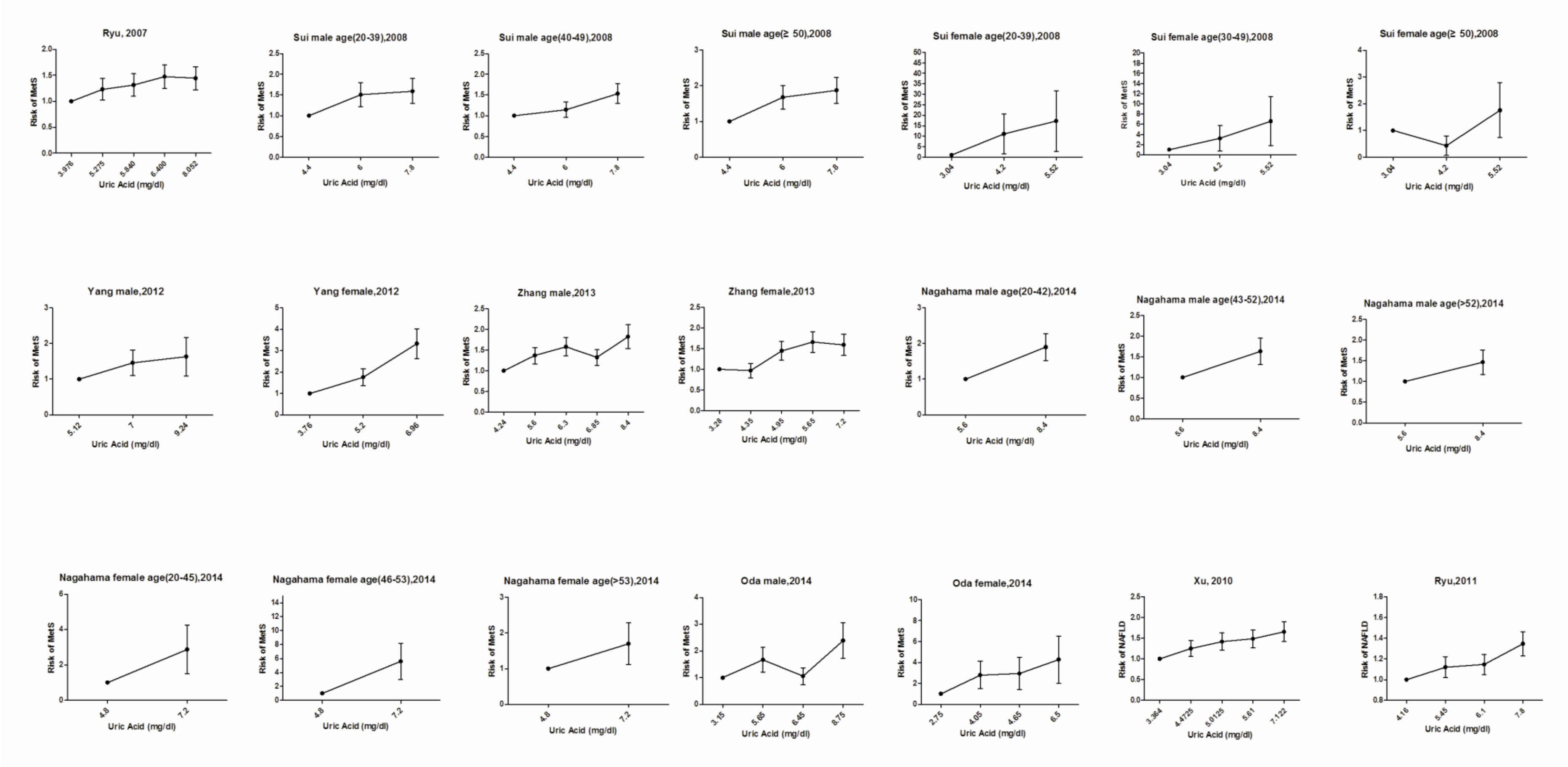

Figure S2 Forest plot of association between serum uric acid and individual metabolic syndrome components in prospective studies

Risks were evaluated per standard deviation of uric acid elevation

A represents the pooled uric acid-obesity association; B represents the uric acid- hyperlipemia association; C represents the uric acid-low-HDL-C association; D represents the uric acid-hypertension association; E represents the uric acid-hyperglycemia association.

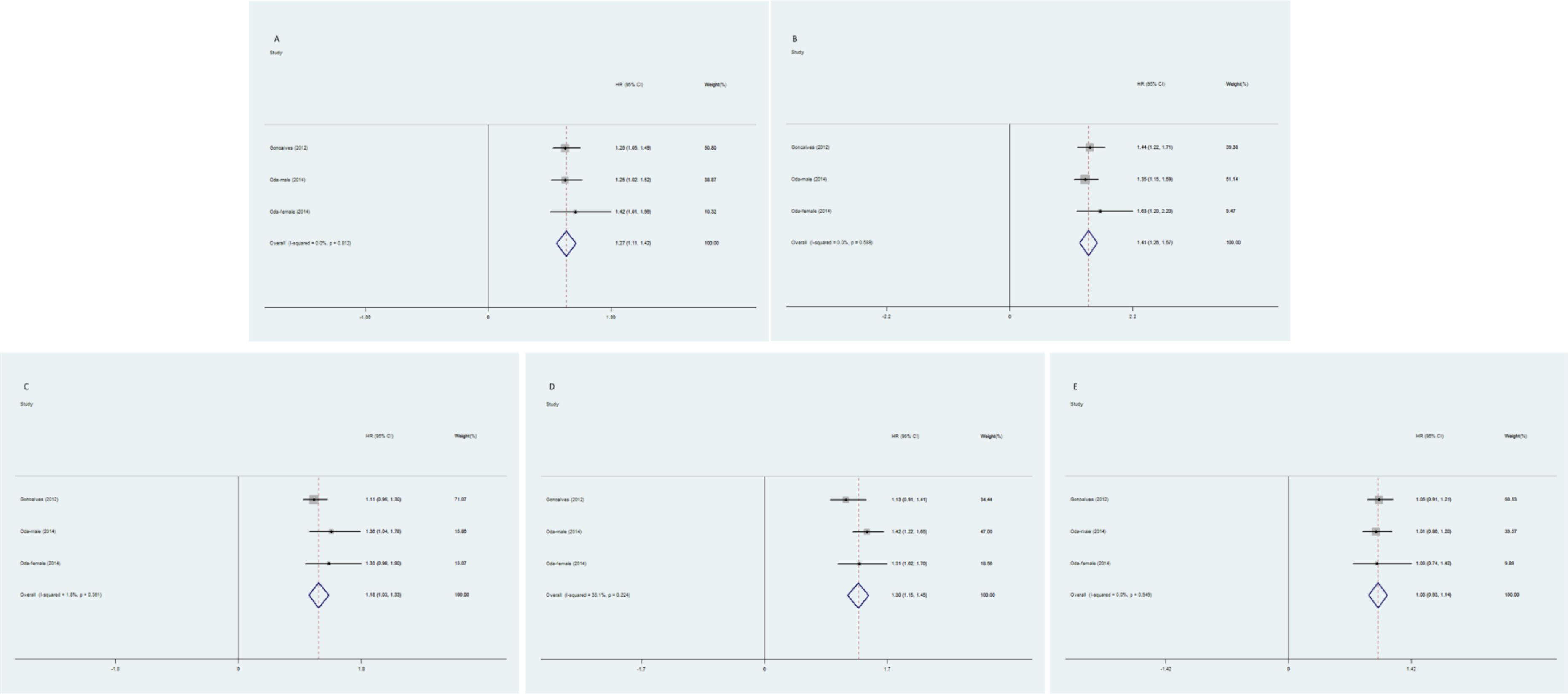

Figure S3 Forest plot of dose-response association between serum uric acid and metabolic syndrome/non-alcoholic fatty liver disease incidence in normouricemic subjects

A represents serum uric acid-metabolic syndrome association; B represents serum uric acid-non-alcoholic fatty liver disease association.

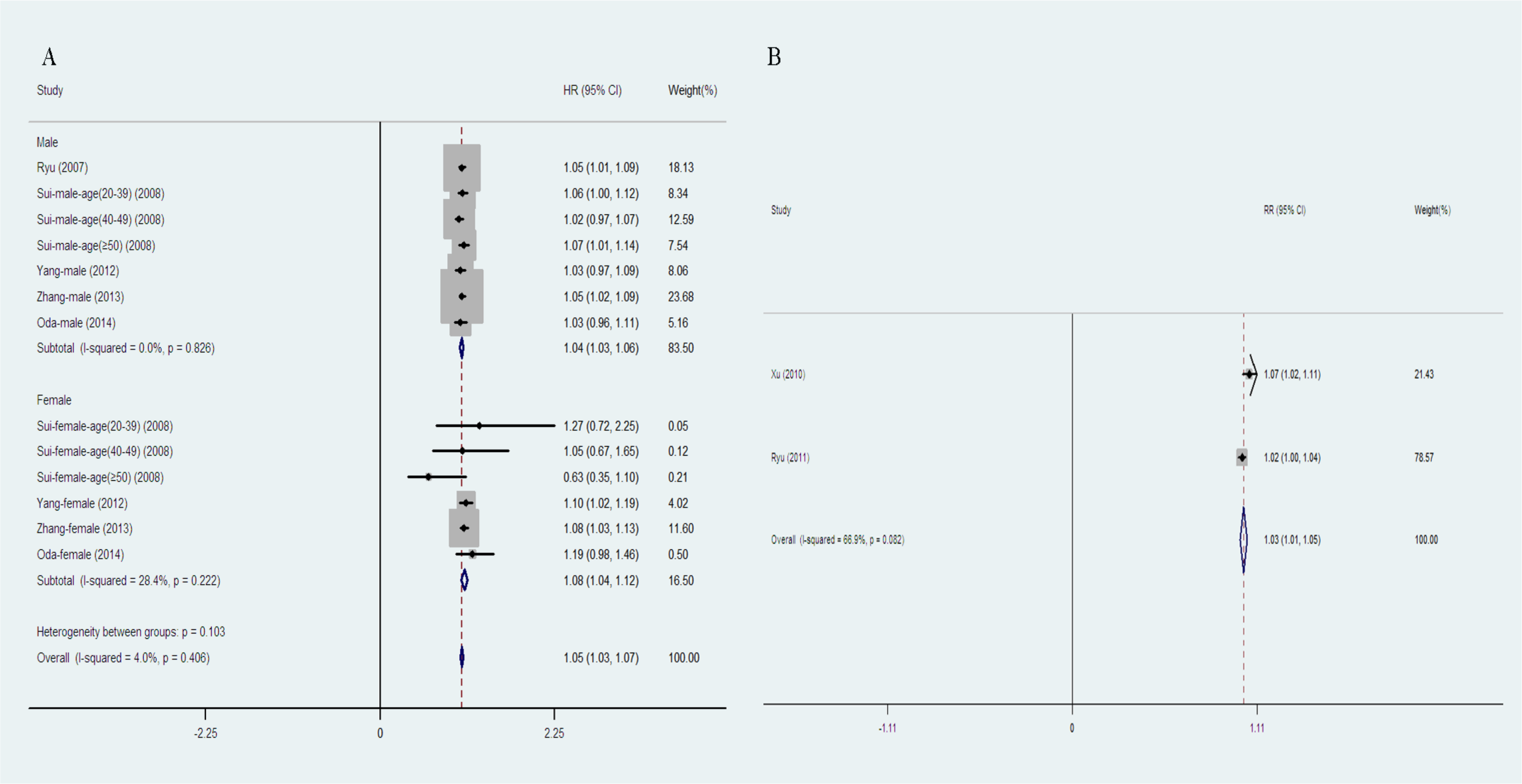

Figure S4 Sensitivity analysis of dose-response association between serum uric acid elevation and risk of metabolic syndrome

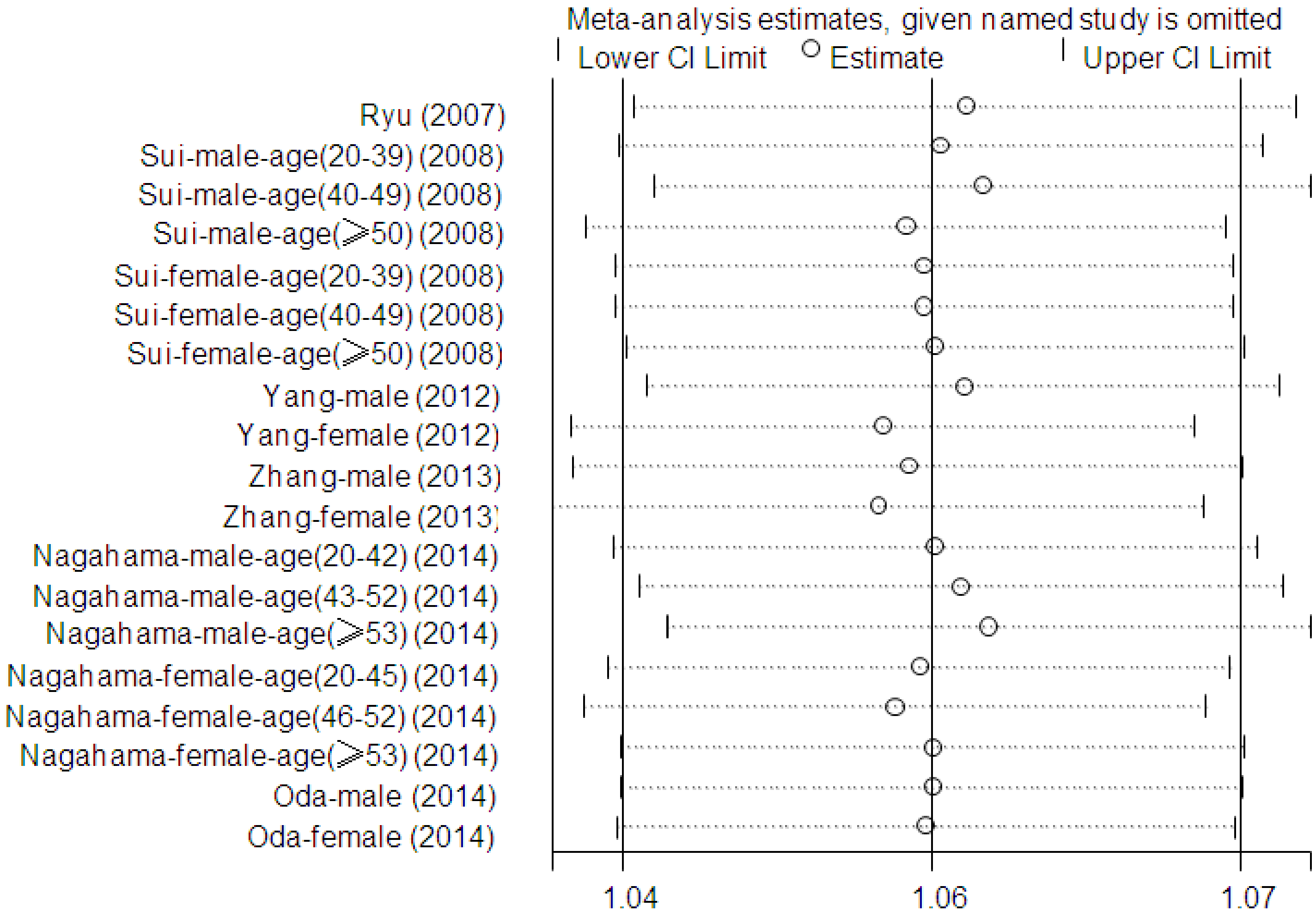

Figure S5 Cumulative meta-analysis of dose-response effect of uric acid elevation on metabolic syndrome risk in prospective studies

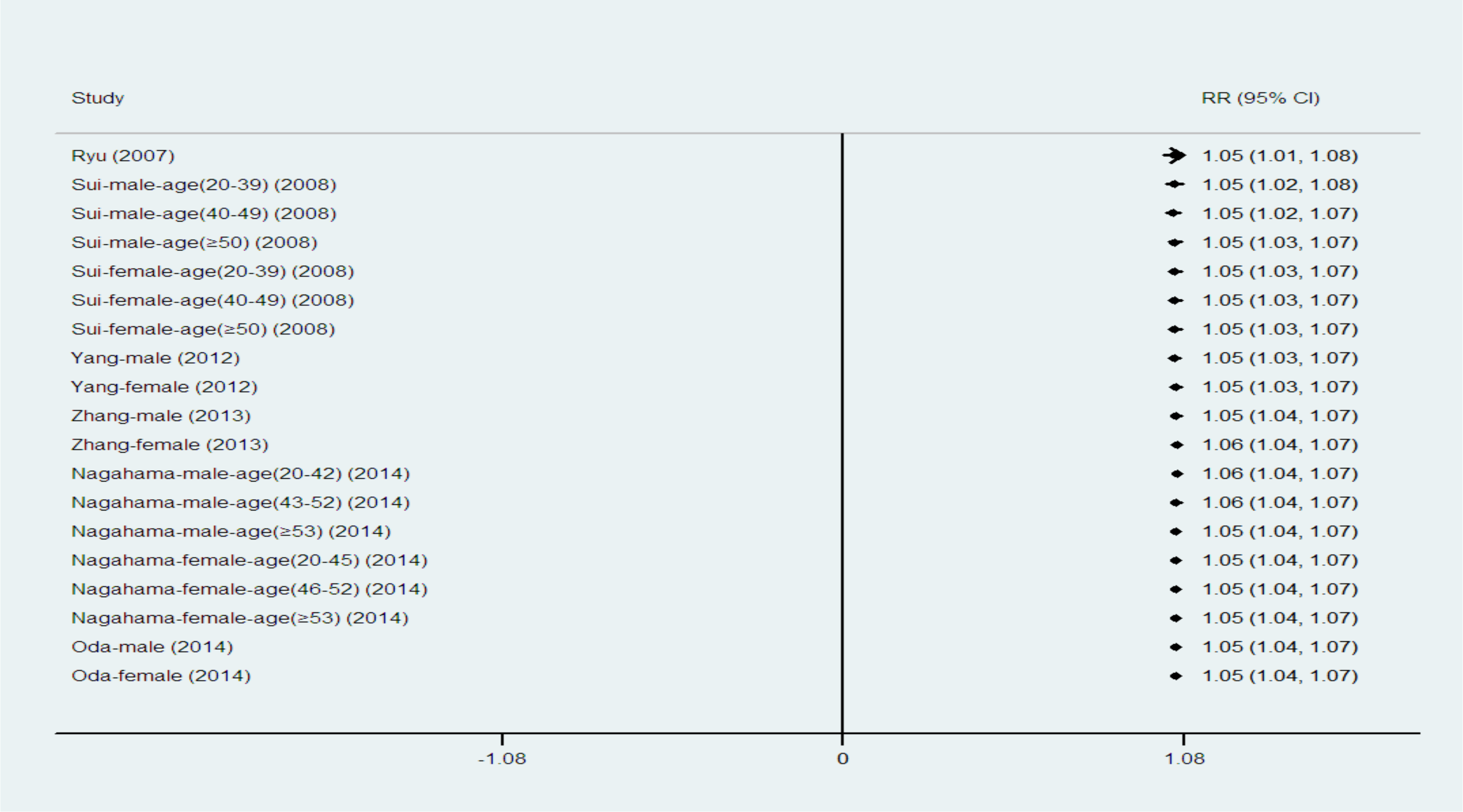

Figure S6 Funnel plot for publication bias in eight studies reporting adjusted hazard ratios of hyperuricemia associated with incident metabolic syndrome

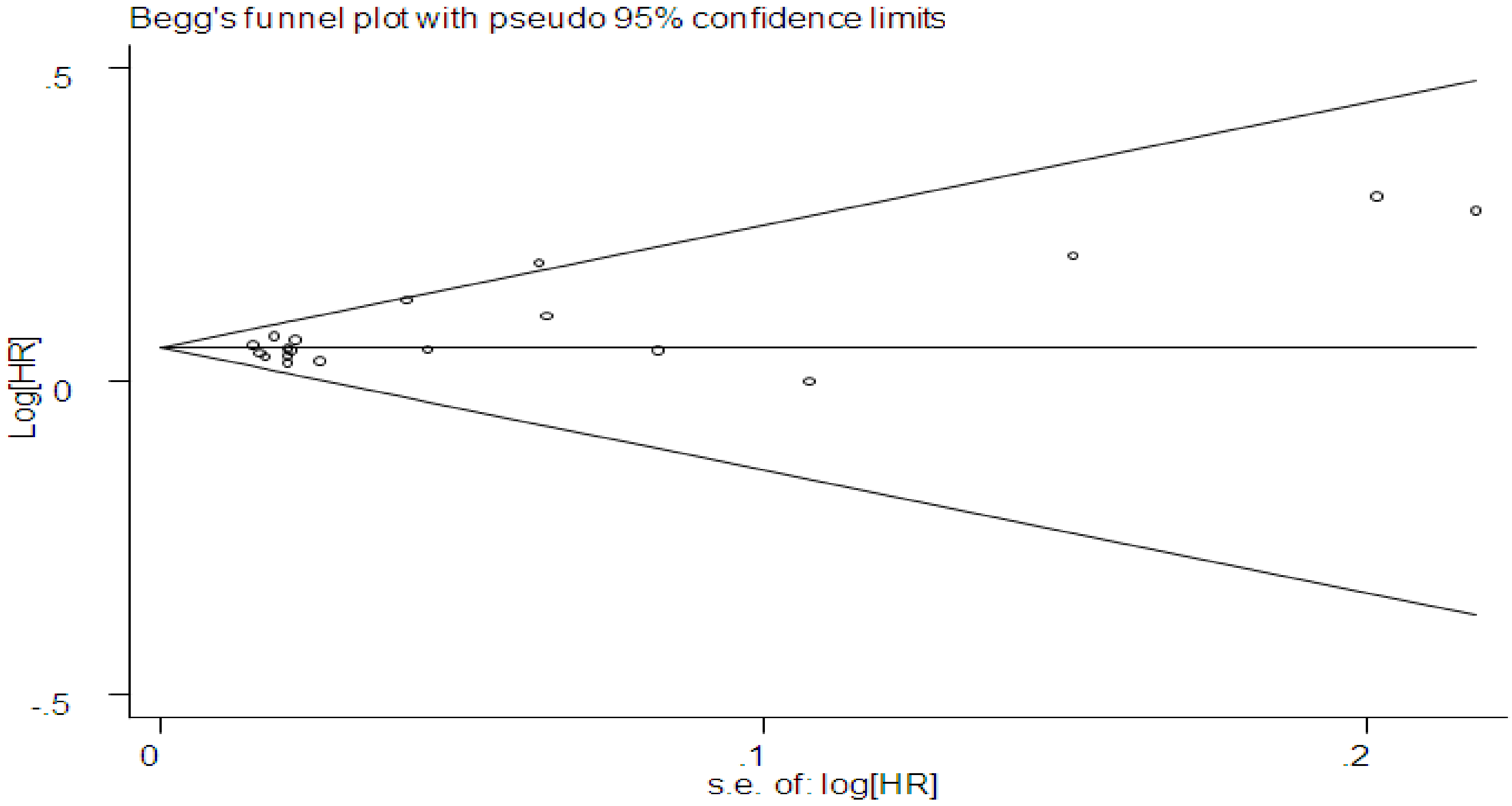

PRISMA 2009 Flow Diagram

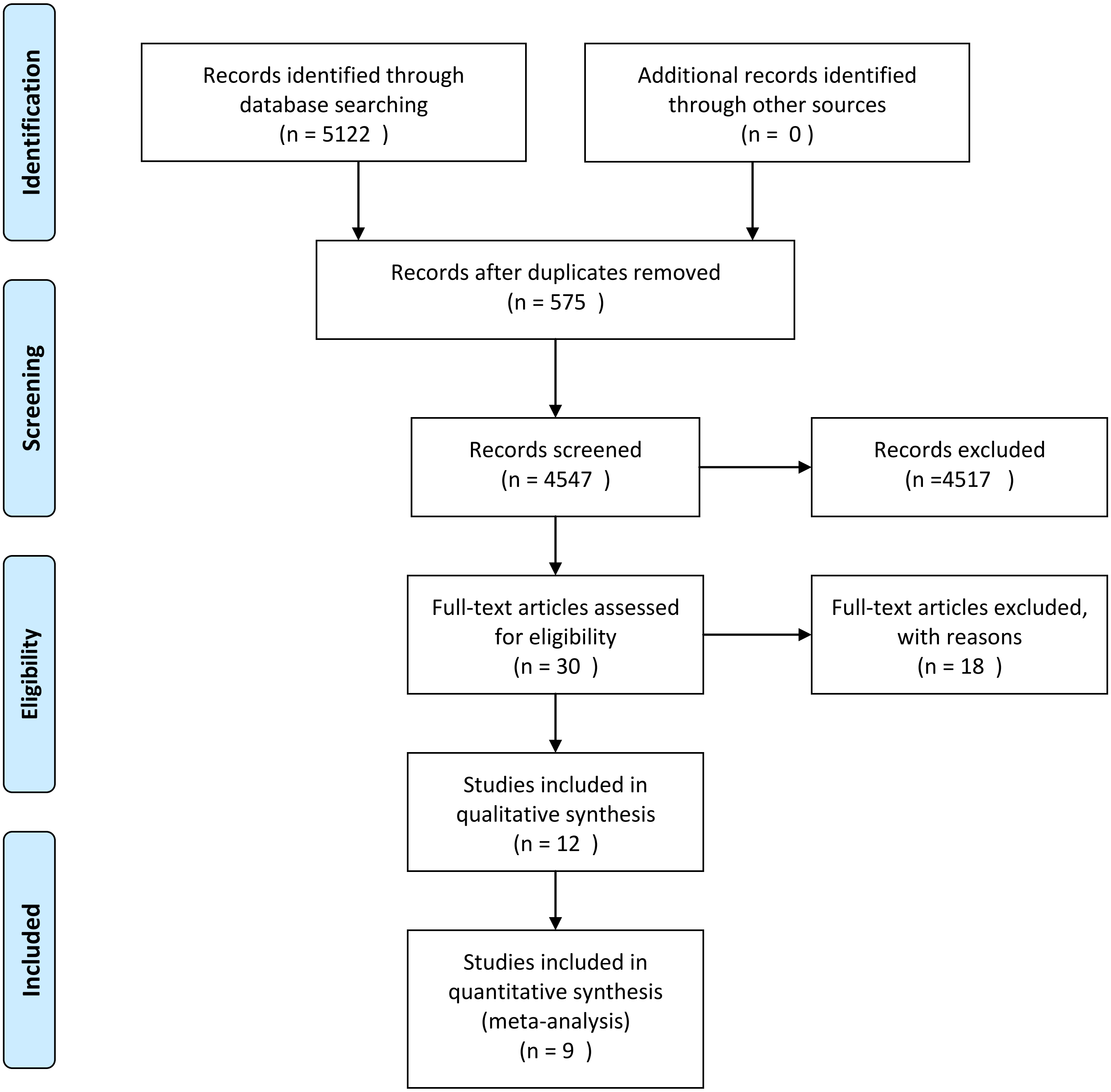

From: Moher D, Liberati A, Tetzlaff J, Altman DG, The PRISMA Group (2009). *P*referred *R*eporting *I*tems for *S*ystematic Reviews and *M*eta-*A*nalyses: The PRISMA Statement. PLoS Med 6(6): e1000097. doi:10.1371/journal.pmed1000097

For more information, visit www.prisma-statement.org.

PRISMA Checklist

Section/topic	#	Checklist item	Reported on page #
**TITLE**			
Title	1	Meta-analysis	1
**ABSTRACT**			
Structured summary	2	Provide a structured summary including, as applicable: background; objectives; data sources; study eligibility criteria, participants, and interventions; study appraisal and synthesis methods; results; limitations; conclusions and implications of key findings; systematic review registration number.	2
**INTRODUCTION**			
Rationale	3	Describe the rationale for the review in the context of what is already known.	3
Objectives	4	Provide an explicit statement of questions being addressed with reference to participants, interventions, comparisons, outcomes, and study design (PICOS).	4
**METHODS**			
Protocol and registration	5	Indicate if a review protocol exists, if and where it can be accessed (e.g., Web address), and, if available, provide registration information including registration number.	4-5
Eligibility criteria	6	Specify study characteristics (e.g., PICOS, length of follow-up) and report characteristics (e.g., years considered, language, publication status) used as criteria for eligibility, giving rationale.	5
Information sources	7	Describe all information sources (e.g., databases with dates of coverage, contact with study authors to identify additional studies) in the search and date last searched.	4-5
Search	8	Present full electronic search strategy for at least one database, including any limits used, such that it could be repeated.	4
Study selection	9	State the process for selecting studies (i.e., screening, eligibility, included in systematic review, and, if applicable, included in the meta-analysis).	5
Data collection process	10	Describe method of data extraction from reports (e.g., piloted forms, independently, in duplicate) and any processes for obtaining and confirming data from investigators.	5-6
Data items	11	List and define all variables for which data were sought (e.g., PICOS, funding sources) and any assumptions and simplifications made.	5
Risk of bias in individual studies	12	Describe methods used for assessing risk of bias of individual studies (including specification of whether this was done at the study or outcome level), and how this information is to be used in any data synthesis.	6-7
Summary measures	13	State the principal summary measures (e.g., risk ratio, difference in means).	6-7
Synthesis of results	14	Describe the methods of handling data and combining results of studies, if done, including measures of consistency (e.g., I^2) for each meta-analysis.	6-7

PRISMA Checklist

Section/topic	#	Checklist item	Reported on page #
Risk of bias across studies	15	Specify any assessment of risk of bias that may affect the cumulative evidence (e.g., publication bias, selective reporting within studies).	6-7
Additional analyses	16	Describe methods of additional analyses (e.g., sensitivity or subgroup analyses, meta-regression), if done, indicating which were pre-specified.	6-7
**RESULTS**			
Study selection	17	Give numbers of studies screened, assessed for eligibility, and included in the review, with reasons for exclusions at each stage, ideally with a flow diagram.	7
Study characteristics	18	For each study, present characteristics for which data were extracted (e.g., study size, PICOS, follow-up period) and provide the citations.	7-8
Risk of bias within studies	19	Present data on risk of bias of each study and, if available, any outcome level assessment (see item 12).	8-11
Results of individual studies	20	For all outcomes considered (benefits or harms), present, for each study: (a) simple summary data for each intervention group (b) effect estimates and confidence intervals, ideally with a forest plot.	8-11
Synthesis of results	21	Present results of each meta-analysis done, including confidence intervals and measures of consistency.	8-11
Risk of bias across studies	22	Present results of any assessment of risk of bias across studies (see Item 15).	8-11
Additional analysis	23	Give results of additional analyses, if done (e.g., sensitivity or subgroup analyses, meta-regression [see Item 16]).	11
**DISCUSSION**			
Summary of evidence	24	Summarize the main findings including the strength of evidence for each main outcome; consider their relevance to key groups (e.g., healthcare providers, users, and policy makers).	11-15
Limitations	25	Discuss limitations at study and outcome level (e.g., risk of bias), and at review-level (e.g., incomplete retrieval of identified research, reporting bias).	15
Conclusions	26	Provide a general interpretation of the results in the context of other evidence, and implications for future research.	15
**FUNDING**			
Funding	27	Describe sources of funding for the systematic review and other support (e.g., supply of data); role of funders for the systematic review.	16

From: Moher D, Liberati A, Tetzlaff J, Altman DG, The PRISMA Group (2009). Preferred Reporting Items for Systematic Reviews and Meta-Analyses: The PRISMA Statement. PLoS Med 6(6): e1000097. doi:10.1371/journal.pmed1000097

For more information, visit: **www.prisma-statement.org**.

Table S1 Search strategy for literatures about the association of uric acid with metabolic syndrome and non-alcoholic fatty liver disease

Pubmed	Search strings	Results
1	uric acid [MeSH Terms]	15003
2	urate [MeSH Terms]	15003
3	gout [MeSH Terms]	7963
4	hyperuricemia [MeSH Terms]	1558
5	UA [Text Word]	3206
6	1 OR 2 OR 3 OR 4 OR 5	22800
7	metabolic syndrome [Text Word]	26505
8	syndrome X [Text Word]	19588
9	insulin resistance syndrome [Text Word]	1404
10	MetS [Text Word]	3975
11	7 OR 8 OR 9 OR 10	29707
12	non-alcoholic fatty liver disease [Text Word]	3400
13	non-alcoholic steatohepatitis [Text Word]	1079
14	steatosis [Text Word]	6297
15	NAFLD [Text Word]	3134
16	NASH [Text Word]	2199
17	11 OR 12 OR 13 OR 14 OR 15	10103
18	6 AND 11	4578
19	6 AND 17	2250
20	18 OR 19	2328
EMBASE		
1	'uric'/exp OR uric AND acid	12258

2	'uric acid'/exp OR 'uric acid'	12251
3	'urate'/exp OR 'urate'	2832
4	'UA'/exp OR 'UA'	6603
5	'hyperuricemia'/exp OR 'hyperuricemia'	4007
6	'gout'/exp OR 'gout'	5063
7	1 OR 2 OR 3 OR 4 OR 5 OR 6	21259
8	'metabolic syndrome'/exp OR 'metabolic syndrome'	19756
9	'syndrome X'/exp OR 'syndrome X'	15971
10	'insulin resistance syndrome '/exp OR 'insulin resistance syndrome '	15706
11	'MetS'/exp OR 'MetS'	3948
12	8 OR 9 OR 10 OR 11	22405
13	'non-alcoholic fatty liver disease '/exp OR 'non-alcoholic fatty liver disease '	4742
14	'non-alcoholic steatohepatitis '/exp OR 'non-alcoholic steatohepatitis'	4623
15	' NAFLD '/exp OR ' NAFLD'	2630
16	' NASH '/exp OR ' NASH'	3733
17	13 OR 14 OR 15 OR 16	7296
18	7 AND 12	1122
19	7 AND 17	139
20	18 OR 19	1235
ISI		
1	Topic=('uric acid')	17922

2	Topic=('urate')	4683
3	Topic=('hyperuricemia')	3287
4	Topic=('gout')	4589
5	Topic=('UA')	5288
6	1 OR 2 OR 3 OR 4 OR 5	27631
7	Topic=('metabolic syndrome')	37193
8	Topic=('syndrome X')	1995
9	Topic=('insulin resistance syndrome')	2378
10	Topic=('MetS')	4045
11	7 OR 8 OR 9 OR 10	40730
12	Topic=('non-alcoholic fatty liver disease')	1932
13	Topic=('non-alcoholic steatohepatitis')	1122
14	Topic=('NAFLD')	3283
15	Topic=('NASH')	8810
16	12 OR 13 OR 14 OR 15	11827
17	6 AND 11	1514
18	6 AND 16	112
19	17 OR 18	1559

Table S2 Check List for Quality Assessment and Scoring of Nonrandomized Studies

Check List
Selection
1. How representative was the selected group in comparison with the general community population? (if yes, one star; no star if the participants were selected or selection of group was not described)
2. How representative was the group with elevated SUA level in comparison with the group within normal range? (if drawn from the same community, one star; no star if drawn from a different source or selection of group was not described)
3. Ascertainment of high risk group in exposure of high SUA concentration (if yes, one star)
4. Demonstration that the disease (MetS/NAFLD) outcome was not present at start of study (if yes, one star)
Comparability
5. Comparison was controlled for age and gender (if yes, one star; no star was assigned if the two groups differed)
6. Comparison was controlled for alcohol intake, cigarette smoking, and baseline MetS components (one star was assigned as if two or more of these three characteristics were controlled for; no star was assigned if one or less characteristic was controlled for)
Outcome assessment
7. Clearly defined disease (MetS/NAFLD) outcome by certain criteria (yes, one star for information ascertained in literature; no star if this information was not reported)
8. Adequate duration of follow-up for observation of ensuing disease (MetS/NAFLD) outcome (one star if duration of follow-up $\geqslant$4 year)
9. Adequacy of follow-up of cohorts (one star if follow-up rate > 90%)

Abbreviations: MetS, metabolic syndrome; NAFLD, non-alcoholic fatty liver disease; SUA, serum uric acid.

Table S3 Quality assessment of the studies included in the meta-analysis by NOS*

NOS scale	Ryu et al. 2007[31]	Sui et al. 2008[32]	Yang et al. 2012[34]	Goncalves et al 2012[35]	Zhang et al. 2013[19]	Nagahama et al. 2014[38]	Oda et al. 2014[40]	Xu et al 2010 [42]	Ryu et al 2011 [33]
**A Selection (maximum 4)**									
**1.Representativeness of general community population**	1	1	1	1	1	1	1	1	1
**2.Subjects with SUA elevation were drawn from the same community with participants within normal UA range**	1	1	1	1	1	1	1	1	1
**3.Ascertainment the exposure of high SUA level**	1	1	1	1	1	1	1	1	1
**4.MetS/NAFLD was not present at baseline**	1	1	1	1	1	1	1	1	1
**B Comparability (maximum 2)**									
**5.Controlled for age and gender**	1	1	1	1	1	1	1	1	1
**6.Controlled for alcohol intake, cigarette smoking and baseline MetS components**	0	1	0	1	1	1	1	1	1
**C Outcome (maximum 3)**									
**7. Clearly defined MetS/NAFLD outcome by**	1	1	1	1	1	1	1	1	1

**certain criteria**									
**8. Adequate duration of follow-up (⩾4 years)**	0	1	1	1	0	1	0	0	1
**9. Adequacy of follow-up rate (>90%) of cohorts**	1	0	0	0	0	1	0	1	0
**Total scores (maximum 9)**	7	8	7	8	7	9	7	8	8

* NOS: Newcastle-Ottawa Scale

"1" meant the study was corresponded to the NOS criteria, "0" meant the study wasn't corresponded to the NOS criteria

Abbreviations: MetS, metabolic syndrome; NAFLD, non-alcoholic fatty liver disease; SUA, serum uric acid.

Table S4 Definition of MetS and its Related Components in Enrolled Studies

Author, publication(ref)	Obesity	Hypertriglyceridemia	low HDL-C	Hyperglycemia	Hypertension	Diagnostic criteria	MetS definition
Ryu et al. 2007[31]	BMI >25 kg/m^2	TG >150mg/dL	HDL-C<40mg/dL	FBG⩾110mg/dL	SBP⩾130mmHg and/or DBP⩾85mmHg	Three of the five criteria were grounds for definition	NCEP-ATP-III on the Asia-Pacific criteria [59]
Sui et al. 2008[32]	WC >102cm for men<br>WC >88cm for women	TG >150mg/dL	HDL-C<40mg/dL	FBG⩾110mg/dL or history of physician-diagnosed diabetes	SBP⩾130mmHg and/or DBP⩾85mmHg or history of physician-diagnosed hypertension	Three of the five criteria were grounds for definition	AHA/NHLBI criteria [57]
Yang et al. 2012[34]	WC >90cm for men<br>WC>80cm for women	TG >150mg/dL	HDL-C<40mg/dL for men;<br>HDL-C<50mg/dL for women;<br>or medication for improving HDL-C	FBG⩾100mg/dL or medication for anti-hyperglycemia	SBP⩾130mmHg and/or DBP⩾85mmHg or the medication of anti-hypertension	Three of the five criteria were grounds for definition	Joint Interim criteria [56]
Goncalves et al. 2012 [35]	WC >102cm for men<br>WC>88cm for women	TG >150mg/dL, and/or drug treatment for elevated TG	HDL-C<40mg/dL for men;<br>HDL-C<50mg/dL for women;<br>or medication for improving HDL-C	FBG⩾100 mg/dL or medication for anti-hyperglycemia	SBP⩾130mmHg and/or DBP⩾85mmHg or the medication of anti-hypertension	Three of the five criteria were grounds for definition	Joint Interim criteria [56]
Zhang et al.	WC>90cm for	TG >150 mg/dL	HDL-C<40mg/dL	FBG⩾100mg/dL	SBP⩾130mmHg	Central obesity	IDF criteria [58]

2013 [19]	men<br>WC>80cm for women	or medication for anti-hyperlipidemia	for men;<br>HDL-C<50mg/dL for women	or medication for anti-hyperglycemia	and/or<br>DBP⩾85mmHg or the medication of anti-hypertension	plus any other two abnormalities	
Nagahama et al. 2014[38]	WC⩾85cm for men<br>WC⩾90cm for women	TG >150mg/dL or the medication for anti-hyperlipidemia	Low HDL-C:<br><40 mg/dL, and/or medication for antidyslipidemia	FBG⩾110 mg/dL or medication for anti-hyperglycemia	SBP⩾130mmHg and/or<br>DBP⩾85mmHg or history of physician-diagnosed hypertension	Central obesity plus any other two abnormalities	Japanese criteria [60]
Oda et al. 2014[40]	WC⩾90cm for men<br>WC⩾80cm for women	TG >150 mg/dL	HDL-C<40mg/dL for men;<br>HDL-C<50mg/dL for women;<br>or medication for improving HDL-C	FBG⩾100 mg/dL or medication for anti-hyperglycemia	SBP⩾130mmHg and/or<br>DBP⩾85mmHg or the medication of anti-hypertension	Three of the five criteria were grounds for definition	AHA/NHLBI criteria [57]

Abbreviations: BMI, body mass index; FBG, fasting blood glucose; DBP, diastolic blood pressure; HDL-C, high density lipoprotein cholesterol; IDF, International Diabetes Federation; MetS, metabolic syndrome; NCEP-ATP-III, National Cholesterol Education Program Adult Treatment Panel III; SBP, systolic blood pressure; SUA, serum uric acid; TG, triglyceride; WC, waist circumference.
